# Awareness of alcohol as a risk factor for cancer is associated with public support for alcohol policies

**DOI:** 10.1186/s12889-018-5581-8

**Published:** 2018-06-04

**Authors:** Sarah Bates, John Holmes, Lucy Gavens, Elena Gomes de Matos, Jessica Li, Bernadette Ward, Lucie Hooper, Simon Dixon, Penny Buykx

**Affiliations:** 1 University of Sheffield, 30 Regent Street, Sheffield, S1 4DA UK; 2 Institut für Therapieforschung, Munich, Germany; 3 Monash University, Bendigo, 3550 Australia; 4 Cancer Research UK, London, UK; 5 dosomething.org, New York, USA

**Keywords:** Cancer, Alcohol, Policy, Support

## Abstract

**Background:**

Globally, alcohol is causally related to 2.5 million deaths per year and 12.5% of these are due to cancer. Previous research has indicated that public awareness of alcohol as a risk factor for cancer is low and this may contribute to a lack of public support for alcohol policies. The aim of this study was to investigate the relationship between awareness of the alcohol-cancer link and support for a range of alcohol policies in an English sample and policy context.

**Methods:**

A cross-sectional survey of 2100 adult residents in England was conducted in which respondents answered questions regarding awareness of the link between alcohol and cancer and support for 21 policy proposals. Principal component analysis (PCA) was used to reduce the 21 policy proposals down to a set of underlying factors. Multiple regression analyses were conducted to estimate the relationship between awareness of the alcohol-cancer link and each of these policy factors.

**Results:**

Thirteen per cent of the sample were aware of the alcohol-cancer link unprompted, a further 34% were aware when prompted and 53% were not aware of the link. PCA reduced the policy items to four policy factors, which were named *price and availability*, *marketing and information*, *harm reduction* and *drink driving*. Awareness of the alcohol-cancer link unprompted was associated with increased support for each of four underlying policy factors: price and availability (Beta: 0.06, 95% CI: 0.01, 0.10), marketing and information (Beta: 0.05, 95% CI: 0.00, 0.09), harm reduction (Beta: 0.09, 95% CI: 0.05, 0.14), and drink driving (Beta: 0.16, 95% CI: 0.11, 0.20).

**Conclusions:**

Support for alcohol policies is greater among individuals who are aware of the link between alcohol and cancer. At the same time, a large proportion of people are unaware of the alcohol-cancer link and so increasing awareness may be an effective approach to increasing support for alcohol policies.

## Background

The global burden of illness and injury from alcohol consumption is high: alcohol is causally related to over 60 major health conditions, is estimated to be responsible for 4.5% of the global burden of disease and injury and accounts for 2.5 million deaths a year worldwide [1]. Rehm and colleagues have listed the range of negative health states which are currently known to be associated with alcohol consumption [2] showing that, among many other diseases, alcohol consumption plays a causal role in several types of cancer. The burden of alcohol-related harm is borne across society, for example through health, social care, justice and lost productivity costs [3, 4]. For example, in the UK in 2009–10 the cost to the National Health Service alone was £3.5 billion and, although the overall cost to society is difficult to estimate, the most widely cited figure, including crime and loss of productivity, is £21 billion a year [5]. Alcohol policy makers charged with balancing government revenue generation, industry regulation, individual freedom and the burden of alcohol need to prioritise the high levels of alcohol-related harm.

Globally, a range of policies are implemented to reduce alcohol-related harm and promote social wellbeing; for example by altering the drinking context, regulating availability and marketing, providing screening and brief interventions or more intensive treatment for heavier drinkers, protecting those at risk from drinkers’ actions, and enhancing the availability of information about the effects of alcohol [6, 7]. Policies with the strongest evidence of effectiveness and cost-effectiveness are those that increase the price of alcohol, and those that restrict availability and marketing [6, 8]. The evidence that information and education policies reduce alcohol-related harm is weaker, although these approaches may be used to reduce the knowledge deficit and change public opinion on policies that are more effective and cost-effective [8].

Public support for health-behaviour policy in general has an inverse relationship with the intrusiveness and/or restrictiveness of the policy, with people tending to prefer policies that they perceive to impact other people and not themselves [9]. This holds true for alcohol-related policies. Internationally, the most effective policies, such as increasing price and restricting availability tend to be the least supported while those with less evidence of effectiveness, such as education, are better supported [10]. For example, of 10 alcohol policy options presented to 1200 UK adults, self-regulation of alcohol advertising gained the most support, whilst a 20–40% reduction in outlets and a minimum unit price of £1 were the least popular policy options [11]. Furthermore, support for increased tax and earlier closing times declined in Ireland between 2002 and 2010, suggesting falling support for effective policies in that country [12]. The lack of public support may contribute to the limited political enthusiasm for some of the policies with the strongest evidence of effectiveness and cost-effectiveness by decision makers [13]; in short, governments are likely to be sensitive to public attitudes towards policy options [9].

There are several factors that are associated with support for effective alcohol policies. Being female, increasing age and consuming none or lower levels of alcohol, compared to high levels, are associated with higher levels of support for more effective policies [11, 14–17]. A higher level of education is associated with greater support for increasing price [16], promotion of limits and warnings, and controlling public spaces [18], and is associated with lower support for restricting availability and greater law enforcement [16]. However, demographic factors are largely non-modifiable. Modifiable factors such as knowledge have also been associated with support for alcohol policy. For example, knowledge of the domain specific (e.g. impact on crime, impact on health), likely positive outcomes of a policy [11] and awareness that alcohol can cause cancer [19] have been associated with support for alcohol policies. So, awareness of potential negative outcomes of alcohol consumption may be a relevant factor in understanding public support for alcohol polices.

A recent review determined that alcohol is now recognised as a risk factor for seven types of cancer including of the liver, mouth and oropharynx and breast [2] however there is an increasing amount of evidence that alcohol has a casual role in other cancers [20] and as such the list of cancers that are attributed to alcohol may grow. Globally, 12.5% of all alcohol-attributable deaths and 8.6% of alcohol-attributable Disability Adjusted Life Years (DALYs) are associated with cancer [1]. Research supports a linear dose-response relationship with an increase in average alcohol consumption positively associated with an increased risk of cancer [21, 22] and even low levels of alcohol consumption have been associated with a small increase in the absolute risk of some types of cancer [23]. Despite this substantial negative health impact, an earlier analysis of the 2015 English population survey data, on which the analyses in this paper are also based, found low levels of awareness of the link between alcohol and cancer [24] with awareness varying by cancer type, from 18% for breast cancer to 80% for liver cancer. These findings echoed similarly low levels of awareness of the alcohol-cancer link in the UK reported six years earlier [25] and are also consistent with findings from an Australian survey [19].

Awareness that alcohol is a risk factor for cancer has been associated with greater support for alcohol policies in the domains of pricing and taxation, availability, marketing and labelling in Australia [19]. While there has been some research within the North-east of England that has examined the impact of a mass-media campaign on awareness of the link between alcohol and cancer and policy support [26], the authors of the current paper were not able to locate any UK-based research that has directly examined the relationship between awareness of the increased risk of cancer and support for alcohol-related policies. Therefore, the aim of the study was to assess which factors are associated with support for different alcohol policies, including awareness of the alcohol-cancer link, in an English sample using policy options of relevance to current UK policy context.

## Methods

### Recruitment

A cross-sectional online survey of 2100 adults was conducted in England in July 2015. The sample size was determined by a pragmatic judgement and no power calculations were conducted. The survey included items on smoking and drinking behaviour, support for/opposition to alcohol policy options, awareness of health conditions associated with alcohol use, and socio-demographic information. A market research company (Vision One) invited existing panel members aged 18 and over to participate in a survey on ‘health and lifestyle behaviours'. Quota sampling was used to ensure the sample was nationally representative with respect to age, sex, geographic region and education. Of the 11,846 members that were sent an email invitation to participate, 5929 started the survey. Following screening for quotas based on the population distribution of sex (male/female), age (18–19, 20–29, 30–39, 40–49, 50–59, 60+), region (North, Midlands and London/South) and education (no qualifications, below degree level, degree level and above) within England, 2480 eligible respondents commenced the survey, of whom 380 were subsequently excluded due to incomplete or invalid responses. To adjust for under-sampling of respondents without qualifications, sample weights were created with reference to the England and Wales 2011 census data [27] (see Table 1).

**Table 1:** Sociodemographic characteristics of the sample and weights applied (*N* = 2100)

	Unweighted		Weights Applied^a^	
	*N*	%	*N*	%
Age				
18–19	63	3.0	62	3.0
20–29	339	16.1	325	15.5
30–39	351	16.7	332	15.8
40–49	394	18.8	385	18.3
50–59	334	15.9	330	15.7
60+	619	29.5	667	31.8
Gender				
Male	1021	48.6	1030	49.0
Female	1079	51.4	1070	51.0
IMD Quintile				
Least deprived	362	17.2	349	16.6
Low deprivation	356	17.0	350	16.7
Average	430	20.5	426	20.3
High Deprivation	469	22.3	474	22.6
Most Deprived	461	22.0	479	22.8
Qualification				
None	178	8.5	315	15.0
Below degree	1238	59.0	1155	55.0
Above degree	684	32.6	630	30.0

^a^Sample weights were created with reference to the England and Wales 2011 census data to increase distribution fit between the sample and the population regarding level of qualification

### Measures

**Fig. 1:**
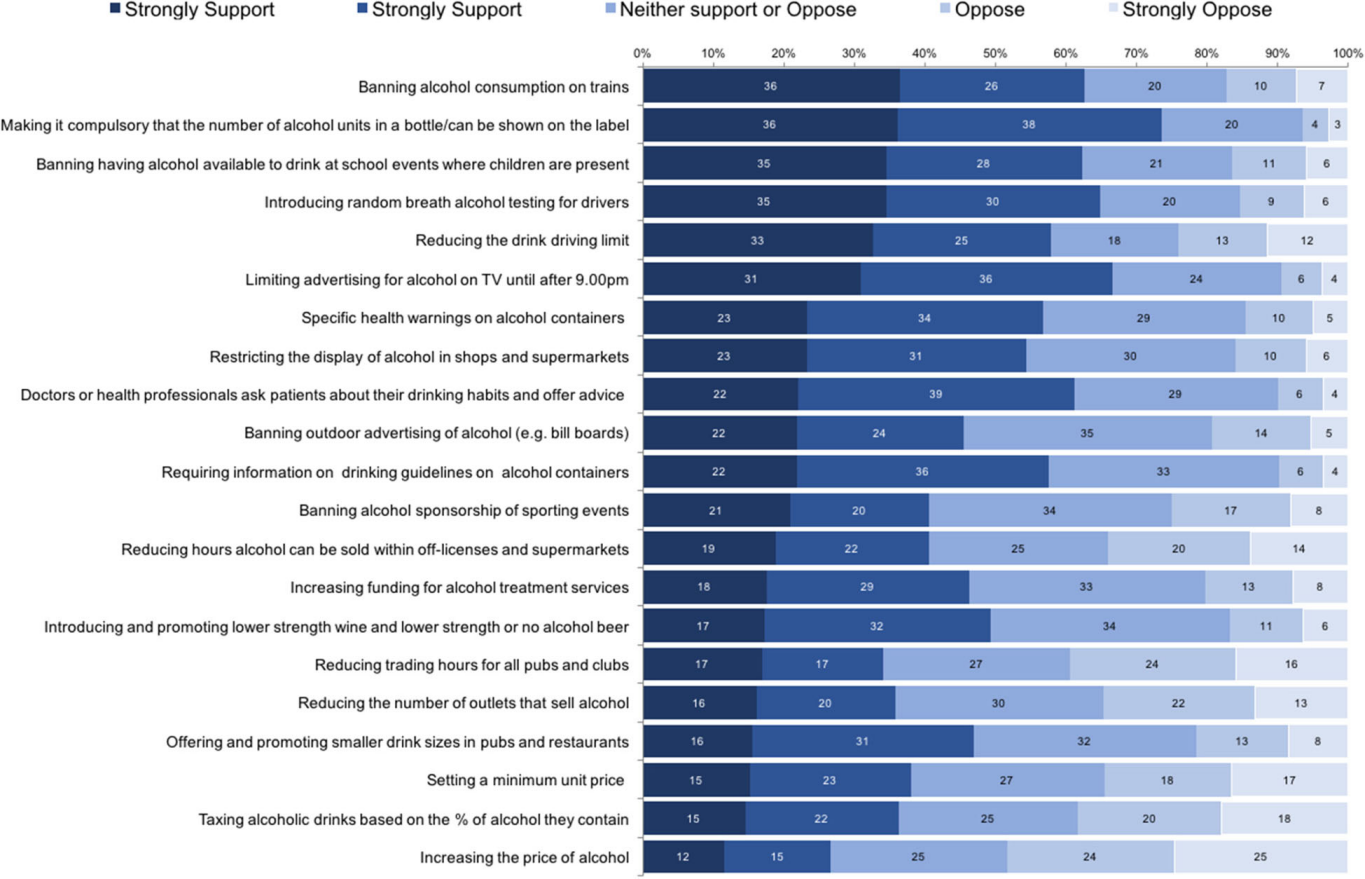
Percentage of participants that support/oppose alcohol policies

To assess support for alcohol policies, respondents were asked ‘*To reduce the problems associated with excessive alcohol use, to what extent would you support or oppose each of the following policies*…?’ followed by a list of 21 alcohol-related policy options (Fig. 1). The question originated from the Australian National Drug Strategy Household survey [28]. Six of the policy options replicated those used in the Australian survey and the remainder were adapted from a recent UK study [16] or devised for this survey (see project report) [29] and covered a range of policy domains (pricing, availability, drink driving counter measures, industry responsibility, labelling, advertising/marketing). Respondents recorded their response on a 5-point Likert scale (strongly oppose, oppose, neither support or oppose, support, strongly support). Awareness of the link between alcohol and cancer was measured firstly in an open question; “*Which, if any, health conditions do you think can result from drinking too much alcohol?*”. Respondents were then presented with a list of health conditions including cancer and asked “*Which, if any, of the following health conditions can result from drinking too much alcohol?*” (yes, no, don’t know). Using these two questions, respondents were categorised into those that listed cancer in the open question (awareness unprompted), those that selected ‘yes’ in the closed questions, but had not already listed cancer in the open question (awareness prompted) and those that did not list cancer when prompted and selected ‘no’ or ‘don’t know’ in the closed section.

Demographic information including age, gender, education (none, below degree and degree or above) and postcode was collected. Postcode data were used to identify 2015 Index of Multiple Deprivation (IMD) quintile, an area-based deprivation measure calculated for 32,844 areas within England, which combines information from 7 weighted domains; income deprivation (weighting factor 22.5%), employment deprivation (22.5%), education, skills and training deprivation (13.5%), health deprivation and disability (13.5%), crime (9.3%), barriers to housing and services (9.3%) and living environment deprivation (9.3%) [30]. IMD quintiles (least deprived, low deprivation, average, high deprivation, most deprived) were based on the national ranking rather than the ranking within the sample. Smoking status was assessed as never-smoker, ex-smoker, or current (occasional or daily) smoker. Alcohol consumption was measured using the three-item consumption scale of the Alcohol Use Disorders Test (AUDIT-C) which assesses past year frequency and quantity of any alcohol consumption and frequency of heavy drinking [31]. AUDIT-C scores were categorised into abstainers (0), lower risk drinkers (1–4), increasing risk drinkers (5–8) and highest risk drinkers (9–12).

### Statistical analyses

Data analyses were conducted using SPSS version 22 for Windows. Analysis involved three stages. Firstly, descriptive analyses were carried out to determine proportion of support in each of the demographic, health behaviour and knowledge categories. Secondly, given the large number of policy items included, Principal Component Analysis (PCA) was conducted to reduce these to fewer factors which underlie patterns of support for individual policy items. The reduction to fewer factors may aid assessment of generalisability of results to other policies not included and avoids increasing the risk of type 1 error by running several analyses. Theoretically there could be correlation between support for one group of policy items and another group so oblique rotation (promax) was used to allow correlation between factors [32]. The PCA generates a score for each individual on each factor. The Kaiser-Meyer-Olkin (KMO) measure was used to assess whether there was an adequate sample size and if KMO values for individual policy items was above the acceptable limit of 0.5 [32]. Bartlett’s test was run to indicate whether correlation between policy items were sufficient for PCA. Kaiser’s criterion, an eigenvalue above one, was used to determine which factors to retain for further analyses. This criterion is reliable in sample sizes of over 250 and when the average communality is 0.6 or larger [32]. Thirdly, to identify predictors of policy support, four linear regression analyses were conducted with the PCA factor scores as dependent variables. Age, age^2^ (entered as continuous variables), gender, IMD quintile (5 categories from least deprived to most deprived), qualifications (no qualifications, qualifications below degree level, degree and above), smoking (never-smoker, ex-smoker, current smoker), alcohol consumption (highest risk drinker, increasing risk, lower risk, abstainer), and cancer awareness (none, prompted, unprompted) were entered as independent variables. Scatter plots of age and support for the four policy factors indicated that the relationship may be quadratic and so age^2^ was included to account for this possibility.

Sensitivity analysis was undertaken by introducing two sets of variables which were not considered in the initial analysis but were highlighted by a reviewer as factors that could impact on policy support. The first was awareness of the link between alcohol and other diseases. There was greater awareness of the link between alcohol and other diseases (heart disease, diabetes, liver disease, high cholesterol or overweight/obesity) than cancer [24]. The second was any history of a cancer diagnosis. These were both included as covariates to examine whether controlling for these impacted on the association between awareness of the alcohol-cancer link and policy support.

## Results

The demographic characteristics are displayed in Table 1. One third (31.4%) of the respondents were current smokers, 24.9% were ex-smokers and 43.7% were non-smokers. The proportions of respondents reporting highest risk, increasing risk, and lower risk drinking were 9.8, 31.5 and 46.8% respectively with 11.9% reporting no alcohol use. When asked about health conditions related to drinking too much, 12.9% listed cancer unprompted and a further 34.3% selected ‘yes’ when cancer was listed as one of a number of potential health conditions resulting from drinking too much. The remaining 52.8% selected ‘no’ or ‘don’t know’.

The PCA analysis revealed that there were correlations between factors of over 0.5 confirming that orthogonal rotation would be inappropriate. The KMO measure indicated an adequate sample size and all KMO values for individual policy items were above the acceptable limit. Bartlett’s test for sphericity was significant indicating the correlations between policy items were sufficient for PCA. The Kaiser criterion was satisfied for the four policy factors. The policy item *Banning alcohol consumption on trains* had a factor loading of below 0.4 on all factors and there was no change to the factor structure when running the PCA without the item so it was removed. The factors explained 65.5% of the variance. Table 2 shows the factor structure and loadings. The four factors identified were labelled *Price and Availability*, *Marketing and Information*, *Harm Reduction* and *Drink Driving* based on the policy items in each factor. The degree of support for each policy option is presented in Fig. 1, ordered from most to least supported.

**Table 2:** Principal Component analysis of 21^a^ alcohol policy items – reduced to four factors

Policy Item	Price and Availability	Marketing and Information	Harm Reduction	Drink driving
Increasing the price of alcohol	.872	−.149	.161	.006
Taxing alcoholic drinks on the basis of the percentage of alcohol they contain	.834	−.128	.207	−.023
Reducing hours alcohol can be sold within off-licenses and supermarkets	.772	.196	−.162	.004
Setting a minimum unit price below which a unit of alcohol cannot be sold	.771	−.027	.177	−.037
Reducing the number of outlets that sell alcohol	.754	.257	−.170	−.056
Reducing trading hours for all pubs and clubs	.753	.157	−.192	.026
Banning outdoor advertising of alcohol such as on bill boards and bus stops	.116	.899	−.117	−.087
Limiting advertising for alcohol on TV until after 9.00 pm	−.050	.833	.082	−.019
Restricting the display of alcohol in shops and supermarkets to dedicated aisles (e.g. not in the entrance)	.134	.773	.036	−.081
Banning alcohol sponsorship of sporting events	.165	.697	−.182	.086
Requiring information on national drinking guidelines on all alcohol containers	−.086	.607	.426	−.068
Specific health warnings on alcohol containers (e.g. like on tobacco packaging)	.012	.580	.334	−.037
Banning having alcohol available to drink at school events where children are present, such as fetes	.057	.553	−.155	.324
Making it compulsory that the number of alcohol units in a bottle or can of alcoholic drink be shown on the label	−.189	.525	.494	.038
Increasing funding for alcohol treatment services	−.036	−.131	.793	−.041
Introducing and promoting lower strength wine and lower strength or no alcohol beer	.316	−.046	.552	.099
Doctors or health professionals ask patients about their drinking habits and, where necessary, offer advice on how to reduce their alcohol consumption	−.065	.393	.502	.045
Offering and promoting smaller drink sizes in pubs and restaurants	.396	.014	.441	.078
Reducing the drink driving limit	−.017	−.101	−.010	.917
Introducing random breath alcohol testing for drivers	−.037	.118	.068	.719

^a^Banning alcohol consumption on trains had a factor loading below 0.4 and when removed from the analysis no change in the factor structure was observed and so was not included

The mean factor score for each variable of interested is displayed in Table 3. Multiple regression analyses (Table 4) demonstrated that awareness of the relationship between alcohol consumption and cancer (unprompted) was significantly associated with support for all policy factors. A significant association was also found for prompted cancer awareness for all policy factors except *drink driving*. Being female and lower levels of alcohol consumption were associated with support for all four policy factors. Alcohol consumption was the strongest predictor of support for *price and availability*, *marketing and information*, and *harm reduction* policies; higher levels of alcohol consumption were associated with lower levels of support excluding the highest risk group. This highest risk group was associated with lower support than the none/low risk groups but greater support than the increasing risk group. Increasing age was the strongest predictor of *drink driving* policies. Education above degree level was associated with greater support for *harm reduction* policies and education below degree level was associated with lower support for *drink driving* policies in comparison to no qualifications. For each of the policy types, the effect size of the association between awareness of the alcohol-cancer link and support for policies was small (Pearson coefficient ranged from 0.06 to 0.15). Of all the policy types, the cancer awareness variable had the largest relative contribution to the degree of support for the *harm reduction* policies. Being an ex-smoker was associated with higher support *for drink driving* policies. Deprivation was not associated with support for any of the policy factors.

**Table 3:** Mean factor based scores by variables of interest

Sample Characteristic	Factor Score							
	Price and Availability		Marketing and Information		Harm Reduction		Drink driving	
	Mean	Sd	Mean	Sd	Mean	Sd	Mean	Sd
Age								
18–34	− 0.08	0.98	− 0.14	0.94	0.07	0.99	−0.11	0.95
35–49	− 0.06	0.97	− 0.08	0.98	0.01	0.98	0.03	0.97
50–64	− 0.01	1.02	0.11	0.98	− 0.01	0.99	0.07	1.05
65+	0.18	1.02	0.13	1.09	−0.09	1.04	0.01	1.03
Gender								
Male	−0.11	1.00	−0.15	1.02	−0.14	1.04	−0.16	1.05
Female	0.11	0.99	0.15	0.96	0.14	0.94	0.16	0.93
IMD quintile								
Least deprived	−0.04	0.93	−0.01	0.97	0.11	0.89	−0.06	0.95
Low deprivation	−0.01	0.99	−0.03	1.02	− 0.05	1.03	−0.00	1.03
Average	0.03	1.00	0.04	0.98	−0.01	0.95	0.07	0.98
High Deprivation	−0.02	1.02	0.01	0.99	−0.02	1.08	−0.03	1.01
Most Deprived	0.02	1.04	−0.02	1.03	− 0.03	1.01	0.02	1.02
Qualification								
None	0.16	1.10	0.14	1.18	−0.14	1.11	0.18	1.06
Below degree	−0.06	0.97	− 0.05	0.96	−0.05	0.98	− 0.06	0.99
Above degree	0.02	1.00	0.02	0.98	0.14	0.97	0.02	0.99
Smoking Status								
Non smoker	0.10	0.99	0.07	0.94	0.09	0.98	0.01	0.99
Ex Smoker	0.01	0.94	0.09	1.00	−0.06	0.97	0.09	0.97
Smoker	−0.14	1.05	−0.15	1.05	−0.07	1.04	−0.08	1.03
Alcohol Consumption								
None	0.86	0.92	0.59	0.92	0.38	0.95	0.36	0.97
Lower risk	0.15	0.92	0.13	0.96	0.07	0.96	0.10	0.95
Increasing risk	−0.35	0.91	−0.27	0.95	− 0.13	0.97	− 0.20	1.00
Highest risk	−0.49	0.95	−0.36	1.02	−0.30	1.17	−0.21	1.06
Cancer knowledge								
None	−0.06	0.99	−0.08	0.99	−0.15	0.98	−0.06	0.98
Prompted	0.04	1.03	0.01	1.03	0.08	1.01	0.02	1.04
Unprompted	0.13	0.96	0.26	0.89	0.36	0.94	0.16	0.97

**Table 4:** Multiple regression analyses – Variables predicting support for alcohol policy factor scores

	Price and Availability				Marketing and Information				Harm Reduction				Drink driving			
	Beta	95% CI		*P* value	Beta	95% CI		*P* value	Beta	95% CI		*P* value	Beta	95% CI		*P* value
Age	−0.09	−0.37	0.19	0.52	0.13	−0.15	0.41	.350	−0.02	−0.32	0.29	.898	*0.49*	*0.20*	*0.79*	*< 0.001*
Age^2	0.18	0.18	0.18	0.21	−0.02	−0.16	0.00	.909	−0.01	−0.16	0.00	.922	*−0.46*	*−0.46*	*0.05*	*< 0.001*
Gender																
Male																
Female	*0.05*	*0.01*	*0.09*	*0.02*	*0.11*	*0.07*	*0.16*	*< 0.001*	*0.09*	*0.04*	*0.13*	*< 0.001*	*0.13*	*0.09*	*0.18*	*< 0.001*
IMD Quintile																
Least deprived																
Low deprivation	0.01	−0.04	0.06	−0.67	−0.01	−0.06	0.05	0.84	−0.05	−0.11	0.00	0.07	0.02	−0.04	0.08	0.47
Average	0.02	−0.03	0.08	.043	0.03	−0.03	0.08	0.39	−0.05	−0.10	0.01	0.11	0.05	−0.01	0.11	0.09
High Deprivation	0.01	−0.05	0.07	0.76	0.02	−0.04	0.07	0.56	−0.04	−0.10	0.01	0.14	0.01	−0.05	0.07	0.84
Most Deprived	0.05	−0.01	0.11	0.10	0.03	−0.03	0.09	0.27	−0.04	−0.10	0.02	0.18	0.04	−0.02	0.10	0.24
Qualification																
None																
Below degree	0.01	−0.05	0.07	0.80	0.00	−0.14	0.14	0.99	0.04	−0.04	0.11	.327	*−0.09*	*−0.16*	*−0.02*	*0.02*
Above degree	0.06	−0.01	0.13	0.09	0.05	−0.02	0.12	0.20	0.11	0.04	0.18	.003	−0.03	− 0.11	0.04	0.38
Smoking Status																
Non smoker																
Ex Smoker	−0.01	− 0.06	0.03	0.55	0.02	−0.03	0.07	0.38	−0.02	−0.07	0.03	.445	*0.06*	*0.01*	*0.11*	*0.02*
Smoker	−0.01	−0.06	0.04	0.67	−0.01	−0.06	0.04	0.58	−0.01	−0.06	0.05	.858	0.01	−0.04	0.06	0.66
Alcohol																
None																
Lower risk	*−0.35*	*−0.42*	*−0.28*	*< 0.001*	*−0.22*	*−0.29*	*−0.14*	*< 0.001*	*−0.15*	*−0.22*	*−0.07*	*< 0.001*	*−0.11*	*−0.19*	*−0.04*	*< 0.001*
Increasing risk	*−0.55*	*−0.62*	*−0.48*	*< 0.001*	*−0.37*	*−0.44*	*−0.30*	*< 0.001*	*−0.24*	*−0.32*	*−0.17*	*< 0.001*	*−0.23*	*−0.30*	*−0.15*	*< 0.001*
Highest risk	*−0.40*	*−0.45*	*−0.34*	*< 0.001*	*−0.26*	*−0.32*	*−0.20*	*< 0.001*	*−0.19*	*−0.25*	*−0.13*	*< 0.001*	*−0.15*	*−0.21*	*−0.09*	*< 0.001*
Alcohol-cancer Awareness																
None																
Prompted	*0.05*	*0.01*	*0.10*	*0.02*	*0.05*	*0.00*	*0.09*	*0.04*	*0.09*	*0.05*	*0.14*	*< 0.001*	*0.04*	−0.01	0.08	*0.12*
Unprompted	*0.06*	*0.01*	*0.10*	*0.01*	*0.11*	*0.06*	*0.15*	*< 0.001*	*0.16*	*0.11*	*0.20*	*< 0.001*	*0.07*	*0.02*	*0.11*	*< 0.001*

p value representing significance are set in italics

### Sensitivity analysis

Respondents that identified a link between alcohol and at least one of heart disease, diabetes, liver disease, high cholesterol or overweight/obesity were compared to those who did not identify any of these links. This awareness was significantly associated with three policy factors *(marketing and information, harm reduction* and *drink driving*), but the inclusion of this variable within the regression had very little impact (change less than or equal to 0.01) on the size of the standardised coefficient of the awareness unprompted of the alcohol-cancer link association. However, for the *marketing and information* factor, the awareness of the alcohol-cancer link only when prompted was reduced to non-significance. Whether or not respondents reported a cancer diagnosis was not significantly associated with policy support and inclusion of these variables did not impact on the coefficients of the awareness of the alcohol-cancer link association.

## Discussion

Awareness of alcohol as a risk factor for cancer is associated with greater support for four different types of policies: *Price and Availability* and *Marketing and Information*, *Harm Reduction* and *Drink Driving*. This study used a three-category variable to distinguish between those that were aware of the alcohol-cancer link unprompted and prompted. This enabled the authors to identify that unprompted cancer awareness is a stronger predictor of support for alcohol policies across all four factors compared with both those who indicated their awareness when prompted or those who were not aware of the risk. Being female and lower levels of alcohol consumption were also both associated with higher levels of support for all policies.

These findings are broadly consistent with previous Australian research, which identified 1) that a similar proportion of the population were aware (either prompted or unprompted) that alcohol is a risk factor for cancer, and 2) that awareness alcohol consumption can cause cancer is associated with support for pricing, availability, marketing and labelling polices [19]. Similarly a British survey that indicated awareness of the link between alcohol and cancer was around 50% in 2015 [33]. We were able to build on this study by examining differential effects of alternative measures of awareness and using PCA in order to examine support for different policy types, thereby increasing the potential generalisability of our results. The findings also reflect results from tobacco-control research in which knowledge of the negative impact of smoking is associated with support for smoking policies [34]. It is likely that the widespread awareness of health risks associated with smoking contributed to the public support for restrictive tobacco policies [35]. In comparison, the awareness of the alcohol-cancer link is low and thus there is a need to increase awareness to allow the public to form informed opinions regarding alcohol policies.

Efforts to improve awareness of the alcohol-cancer link may contribute to decreasing the knowledge deficit. There is some evidence that public campaigns can increase public awareness of the link between alcohol and cancer [24]. In the North-east of England, people who were exposed to a mass-media campaign to raise awareness were more aware of the link between alcohol and cancer than those who had not and support for alcohol policies increased following the campaign [26]. In Denmark, a week-long annual alcohol campaign run over 10 years increased public knowledge of safe drinking limits [36]. Within this study, it is not known whether increased awareness has an impact on support for policies; however, Pechey and colleagues have demonstrated that preferences for policies could be altered if the probable positive outcomes are presented alongside the policy [11]. For example, the popularity of minimum unit pricing policies was much greater (supported by an additional one-fifth of participants) when all probable positive outcomes were presented compared to when no outcomes were presented. A study conducted in New Zealand found that public support for alcohol control policies was maintained in communities exposed to alcohol-related health promotion media campaigns and community-based intervention activities whereas it declined in those communities without any such intervention [37]. Together, these studies suggest that public awareness of the health risks of alcohol consumption can be increased and that increased awareness may have an impact on public support for alcohol policy, particularly where there is a clear description of anticipated policy effects. However, attempts to raise public awareness may be resisted by alcohol-funded organisations as has been reported to have occurred recently in response to warning labels on alcohol products in Canada [38]. Although there is evidence to indicate that increased awareness does not necessarily reduce actual consumption of alcohol [39], this study indicates that awareness is associated with greater support for policies and thus has the potential to reduce alcohol-related harm indirectly through generating an environment where restrictive policies are more likely to be implemented.

This study has limitations; the respondents were recruited from an existing market research panel and therefore membership of the sampling frame is self-selecting and limited to those who have access to, and are confident using, the internet. Furthermore, of the people that received the email, only approximately 50% started the survey and information is not available about those that did not respond so any potential differences between the responders and non-responders is not known. These factors may have generated a selection bias. However, quota sampling was used with the aim of creating a representative sample of England based on age, gender, region, and education level and weights were applied to adjust for differences between the sample and the population in order to maximise the generalisability of the study findings. The analyses focussed on awareness of alcohol as a risk factor for cancer in general and did not examine whether awareness of the risk of certain cancers are stronger or weaker predictors of support for policies, especially as awareness of the role of alcohol as a risk factor varies depending on the type of cancer [40]. Further, the policy items presented were simply descriptive (e.g. ‘increasing the price of alcohol’) and did not detail what the anticipated policy effects might be and for whom. Including likely positive outcomes of a policy has been associated with preferences for alcohol policy and thus this may have impact on outcomes [11] .Support for policy may also be influenced by personal experience. Greater support has been found among those who have experienced alcohol related-harm or alcohol-related disturbance [41] and so those who have experience of alcohol-attributable cancer may report greater support for policy. This may have confounded the association found between awareness and support for policy. Although information about the type of cancer (i.e. whether it was alcohol-attributable) and personal experience of cancer other than a personal diagnosis (i.e. a diagnosis of a friend or family) was not available, the impact of any cancer diagnosis was controlled for in sensitivity analysis and this did not have an impact on the association between alcohol-cancer awareness and policy support. Finally, we cannot be certain that reported support represents actual support however any increase in support may create environment in which these policies are more likely to be implemented.

Whilst the methods used in the PCA were directed by research recommendations [32], alternative methods could be employed relating to, amongst other things, rotation method and factor selection. Several alternative methods were examined, but they did not have an appreciable impact on the findings reported here. Likewise, alternative regression methods could have been employed, however, the distribution of the dependent variables would strongly suggest that linear regression is the most appropriate method, and this approach is commonplace among other analogous studies.

Several potential future research questions arise from this study. The study was cross-sectional. While previous research has demonstrated that alcohol-awareness campaigns can raise awareness of the link between alcohol and cancer [39] and can increase support for alcohol policies in the North-East of England [26] future prospective research could usefully examine whether exposure to information and an increase in awareness, is associated with a change in policy support in a wider population. This would help us to develop a better understanding of how increasing awareness might change public opinion on effective policies that are politically challenging to implement (e.g. minimum unit pricing). Future research could examine the combined effect of increased awareness of the general health risks of alcohol in addition to providing more detailed information about anticipated policy effects. Finally, future research could examine health or other risks not only to the individual but also people close to them as a predictor of policy support. A previous study examining support for restrictions on tobacco found awareness of the potential harm to others strongly predicted support [34] and it may be that a similar relationship exists for alcohol.

## Conclusions

The extent to which any individual supports a government policy is dependent on a range of factors including the behaviour the policy targets (e.g. smoking, alcohol consumption), the type of policy and how intrusive it is (e.g. taxation, regulation), who the policy targets (e.g. children), and the extent to which the individual in question will be affected by the policy. Some predictors of support for policies are modifiable. Awareness, and especially unprompted awareness, of the link between alcohol consumption and cancer may be one such modifiable predictor, given that awareness of the risk is a significant predictor of support for range of alcohol policies. Therefore, improving awareness of the link between alcohol consumption and cancer may increase public support for effective alcohol policies that are otherwise relatively unpopular. These results are useful for policy-makers, because it highlights that understanding of a policy and its context is an important determinant of support and that increasing awareness of the specific harms being addressed may result in greater support for alcohol policies.
